# Orthokeratology in Moderate Myopia: A Study of Predictability and Safety

**DOI:** 10.18502/jovr.v15i2.6739

**Published:** 2020-04-06

**Authors:** Kirti Singh, Mainak Bhattacharyya, Abhishek Goel, Ritu Arora, Nikhil Gotmare, Himshikha Aggarwal

**Affiliations:** Guru Nanak Eye Centre, Maharaja Ranjit Singh Marg, New Delhi, India

**Keywords:** Contact Lens, Overnight Wear, Orthokeratology, Moderate Myopia, Semi-tropical Environments

## Abstract

**Purpose:**

Literature is relatively silent on safety profile and predictability of orthokeratology lenses in terms of myopia correction and prevention of further progression, especially in semi-tropical countries; this study was designed to fill this gap.

**Methods:**

This prospective, intervention case series enrolled 30 eyes of 30 patients with myopia up to –5.5 diopters (D). Patients were randomized into two groups of 15 each; the study group was prescribed overnight orthokeratology (OK) lenses, while the control group used daily wear conventional soft contact lenses. Follow-up examinations were performed after 1 h and 6 h, and then at 1, 7, 15, 30 days, and 4 months post lens wear. Uncorrected visual acuity (UCVA), contrast sensitivity, keratometry, central corneal thickness (CCT), and tear film break up time (TBUT) were evaluated at each follow-up examination.

**Results:**

All patients attained a visual acuity of 0.00 Logarithm of the Minimum Angle of Resolution (logMAR) after one week of lens use, which was maintained throughout the study period. While patients allotted to the study group had a gain of 8.1 Snellen lines (UCVA), those in the control group gained 8.9 lines (BCVA) at the end of follow-up period. In the OK group, cornea showed a flattening of 0.8 D (mean keratometry) after single overnight usage of OK lens and overall flattening of 1.2 D compared to baseline, at the end of four months. The change in contrast sensitivity, corneal endothelial specular count, axial length and tear film status was not significant in either group.

**Conclusion:**

Orthokeratology is an effective and safe modality to correct moderate myopia in motivated young adults. No side effects were encountered after a short-term follow-up in participants who resided in semi-tropical environments.

##  INTRODUCTION

Orthokeratology (OK) is defined as “reduction, modification, or elimination of a refractive error by programmed application of contact lenses.”^[1]^ Its genesis arose following a serendipitous observation by Wesley and Jessen in the 1950s who found spectacle blur experienced by patients after wearing hard contact lenses. This perceived blurring was subsequently traced to lens-induced corneal reshaping, which was then utilized for therapeutic purposes.^[2]^ The method was eventually discontinued due to problems of poor lens fitting, unpredictable results, and safety concerns.^[3,4,5,6]^ This lost science of OK was revived in the late 1980s with the development of reverse-geometry contact lens designs by Wlodyga and Stoyan.^[7]^ Their design permitted more predictable results to be achieved over shorter periods. Further refinement using very high oxygen-permeable materials ushered in an era of overnight lens wear. This overnight OK lens reduced daytime visual aid requirements and came to be known as “accelerated orthokeratology” because of the rapid onset of refractive and corneal topographic changes.^[8]^

The OK lenses work by altering the corneal shape from prolate to oblate, resulting in a reduction in the central corneal curvature. This alteration of shape is a result of the flat lens design, causing a redistribution of corneal epithelium and anterior stromal tissue over the central treatment zone which is 5 to 6 mm in diameter.^[9]^ The central treatment zone is dictated by pupil size, and treatment zone less than mesopic pupil dimensions can adversely affect vision in conditions of low-illumination and low-contrast. The reversibility of the procedure within two to four weeks of discontinuation of OK lens wear is a consequence of regression of corneal parameters.^[10]^

Common issues of concern with OK lens use include the unpredictability of visual gain due to issues of inadequate lens centration, poor contrast with persistent use, and increase in higher-order aberrations (HOA) and safety because of the inherent risk of microbial keratitis.^[11]^ In semi-tropical environments, microbial growth is expected to be more florid, especially in the closed-eye conditions simulated by OK lens wear. These risks underscore the importance of safety profiling of OK lenses in semi-tropical environments. Previous studies have also reported a high rate of keratitis associated with lens use in semi-tropical regions.^[12,13]^ Stringent lens care regimens have made OK a safe overnight treatment modality, but evidence on safety and predictability of OK lens use in semi-tropical environments is relatively scarce; this study was designed to bridge this gap.

##  METHODS

This study was a prospective, interventional trial of OK lens wear in 30 eyes of 30 patients with myopia up to –5.5 diopters (D). This research followed the tenets of the Declaration of Helsinki. Informed consent was obtained from all participants after explanation of the nature and possible consequences of the study. This research was approved by our local Institutional Ethical Committee. Inclusion criteria were: age 18–30 years; myopia up to –5.5 D, and with-the-rule astigmatism of up to –1.5 D or against-the-rule astigmatism up to –0.75 D with keratometry values between 40 and 45 D. Patients with pathological myopia, corneal pathologies such as dry eyes, healed keratitis, ocular surgery, keratoconus and systemic comorbidities such as diabetes mellitus and thyrotoxicosis were excluded from the study. Patients were randomized (using the closed envelope method) into two groups of 15 each: the study group (OK group) was prescribed OK lenses in one eye only and the control group was prescribed daily wear conventional soft contact lenses in both eyes.

The evaluated parameters were cycloplegic refraction, uncorrected visual acuity (UCVA), best-corrected visual acuity (BCVA) using a Snellen chart, corneal topography and keratometry by Orbscan IIz (Bausch & Lomb Technolas Topographer, USA), central corneal thickness (CCT) (Sonomed 300P PacScan; Sonomed Escalon, USA), tear film break up time (TBUT), contrast sensitivity (using FACT charts), corneal endothelial cell count (Nidek CEM 530, Nidek Co Ltd, Japan), and axial length.

The study group was subjected to a trial of reverse geometry lenses, made of Boston XO material with diffusion constant (DK) value 140 (OrthoK Lucid Korea lenses). These light blue colored lenses of 10.6 mm diameter were available in a power range of –0.50 D to –5.0 D with base curves ranging from 8.44 mm (40.00 D) to 7.42 mm (46.00 D). The flat K reading was used to calculate the base curve of the first trial lens.

**Figure 1:**
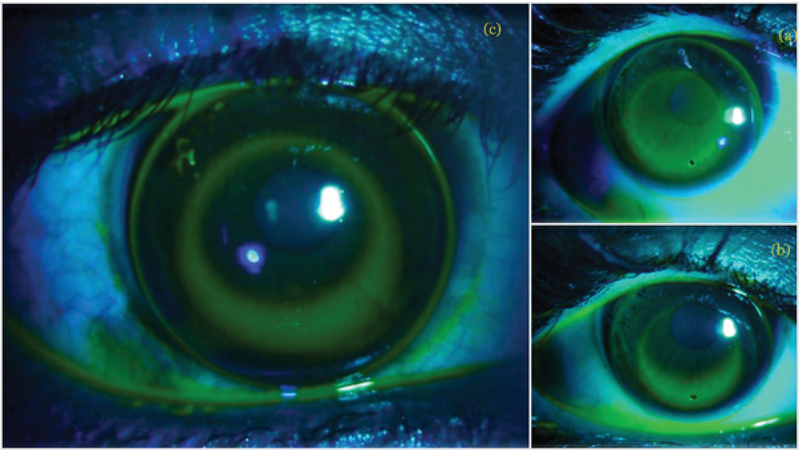
Sequential fitting from steep fit to ideal fit during trial fitting of an orthokeratology patient.

Sequential flattening or steepening was performed in steps of 0.4–0.5 BC until an optimal fluorescein bull's eye pattern was attained [Figure 1]. A movement of 1 mm was the goal for dynamic fit, but a greater emphasis was placed on the static fit. Patients were assessed after 1 h of wear to confirm adaptability and detect early improvements (if any) in visual acuity. The lens was then inserted in one eye, and the patient was admitted overnight to ensure initial comfort and adherence.

UCVA, contrast sensitivity, keratometry values, CCT, and TBUT were evaluated in the morning after the first night of OK wear. The patients were then discharged and asked to resume normal daily activities while using OK lenses during their 8–9 h of night-time sleep. The duration of OK lens usage was determined based on the amount of myopia to be corrected. For mild myopia (–2.0 to –3.5 D), the OK lens was worn every night for three weeks followed by alternate nights. For moderate myopia ( > –3.5D), the lens was worn every night. Subjects were followed-up at days 7, 15, 30, and then monthly for four months.

Subjective assessments of the comfort and tolerability of lens wear were evaluated for each patient at the beginning and end of the study. The criteria used included quality of vision, comfort/difficulty of lens wear, ghosting of images, halos, flares, or difficulties in performing tasks in dim light.

The CL group subjects were prescribed conventional daily wear soft contact lenses (Silk Lens) with a Dk value of 50 and a thickness of 0.1 mm. These patients were not admitted for the first night, but instead were followed-up at 1 h and 6 h of lens wear on days 1, 7, 15, 30, and then monthly until four months. The lens usage was as per requirements, which ranged from 6–9 h per day. Both eyes were prescribed soft lenses, but only one eye was included for the purpose of the study.

### Statistical Analysis

Continuous variables were expressed as means ± standard deviations, and categorical variables were expressed as frequencies and percentages. An independent *t*-test was used to compare the continuous variables between the OK and control groups, whereas a paired *t*-test was used to compare the change of measurement results for paired samples. A Chi-squared test or Fisher Exact test was used to examine differences in categorical variables. Statistically significant differences were defined as *P* < 0.05. Statistical analyses were carried out using the Statistical Package for Social Sciences (SPSS) Version 17.0 software.

##  RESULTS

The OK and control groups were age matched, with the average age being 22.3 ± 2.8 years in the OK group and 22.1 ± 6.8 years in the control group (*P* = 0.9). The sample consisted of 16 females and 14 males. The mean spherical refractive error in the OK group was –3.4 ± 1.3 D (–2.0 to –5.5 D), with a cylindrical error of –0.4 ± 0.5 D at 69 ± 32° (–0.25 to –1.5 D at 15–95°). The mean spherical refractive error in the control group was –3.4 ± 1.03 D (–1.8 to –5.3 D) with mean cylindrical error of –0.2 ± 0.3D at 95 ± 6° (–0.25 to –1.75D at 20–100°). No statistically significant intergroup differences were found in the distribution of refractive errors using the Mann–Whitney test (*P* = 0.351).

### Visual acuity 

**Figure 2:**
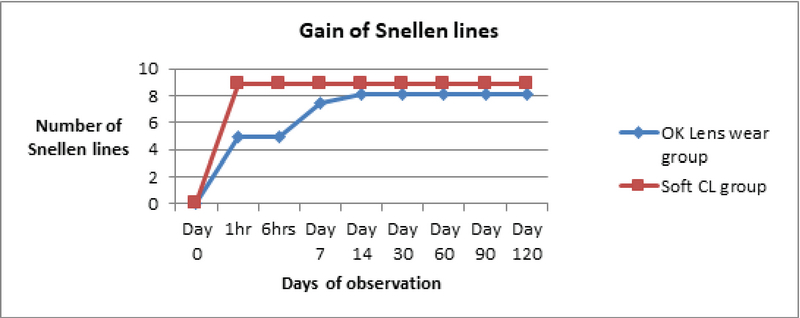
Gain in number of Snellen lines after lens wear. CL, contact lens; OK, Orthokeratology

Mean visual acuities in the OK and control groups at presentation were 1.07 ± 0.15 and 1.040 ± 0.11 Log MAR, respectively (*P* = 0.635). At all subsequent follow-ups, visual acuity measures were taken after the removal of the OK lens in the study group and with the soft lens *in situ* in the control group. Post lens wear visual acuity at 1 h was 0.31 ± 0.3 LogMAR in the OK group versus 0.00 LogMAR in the control group. At day 7, the visual acuities were 0.05 ± 0.1 LogMAR versus 0.00 LogMAR in the OK and control groups, respectively. After that, both groups had a visual acuity of 0.00 LogMAR at all subsequent follow-ups. A statistically significant intergroup difference in visual acuity was seen only at 1 h and 6 h of lens wear (*P* < 0.001) [Figure 2].

In the OK group, the gains in Snellen lines of visual acuity were 4.98 at 1 h, 8.1 lines and the end of follow-up. In the control group, the gains were 8.9 lines at all follow-ups beginning at 1 h. This underscores the fact that OK lens wearers took time to attain good unaided visual acuity, whereas control wearers (the control group) had an instant gain of aided good vision.

Visual acuity of 0.0 LogMAR units (6/6 Snellen) was achieved after one day of overnight wear in patients with mild myopia and by day 7 of overnight wear in patients with moderate myopia. The target of visual acuity of 0.0 LogMAR (Snellen 6/6) was attained in all patients and was maintained for the entire four-month follow-up period. A drop of 1.2 lines in the evening time was documented in the OK group during the first week of the OK lens use.

### Corneal alterations and effect on contrast sensitivity and tear film

**Figure 3:**
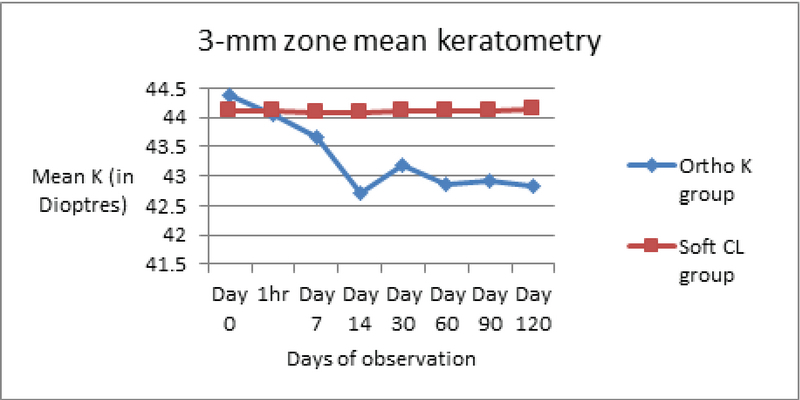
Change in mean keratometry in both groups in 3-mm zone. CL, contact lens; K, keratometry; Ortho K, Orthokeratology.

**Figure 4:**
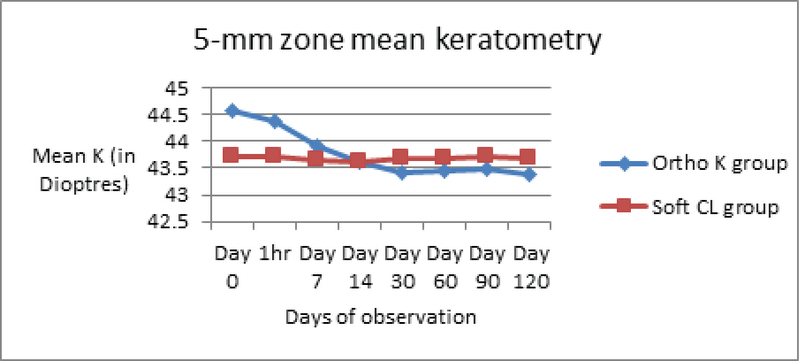
Change in mean keratometry in both groups in 5-mm zone. CL, contact lens; K, keratometry; Ortho K, Orthokeratology.

Corneal alterations were assessed by evaluating changes in corneal topography, pachymetry, and endothelial cell counts. Additional deleterious effects (if any) on contrast sensitivity and tear film dynamics were recorded [Table 1]. Corneal topography exhibited maximal changes in keratometry readings occurring within the first night of OK lens wear. These effects were particularly evident within the first hour of lens wear, reaching a significant level (*P* = 0.034). This change plateaued by 6 h of wear with no further significant change. Over the longitudinal follow-up period, the maximal change in mean Sim K (measured at central 3- and 5-mm central zones) occurred within the initial four weeks of OK lens wear [Figures 3 and 4]. As depicted in Table 1, this change was highly significant at the final follow-up at four months.

**Table 1:** Change in corneal pachymetry, 3-mm and 5-mm zone keratometry, specular count tear film break-up time, axial length and contrast sensitivity in the study (Ortho K) and control groups post lens wear


	**Ortho K group**		**Control group**		**Intergroup comparison**
	**Pre lens wear**	**Post lens use (four months)**	**Pre lens wear**	**Post lens use (four months)**	
Corneal Pachymetry	527 ± 28.4µm	525.8 ± 27.4 µm	532 ± 27.4 µm	530.80 ± 29.07 µm	NS
Keratometry (3-mm zone)	44.37 ± 1.3 D	42.83 ± 1.1 D 1.54 (*P* = 0.000)	44.12 ± 0.9D	44.14 ± 0.9 D	*P = *0.002 Significant
Keratometry (5-mm zone)	44.58 ± 1.2 D	43.38 ± 0.7 D 1.2 (*P* = 0.000)	43.72 ± 1.0 D	43.68 ± 0.9 D	NS
Specular count	2901.40 ± 305.5 cells/mm ${}^{3}$	2894 ± 294.6 cells/mm ${}^{3}$	2772.87 ± 290.3 cells/mm ${}^{3}$	2733.20 ± 249.6 cells/mm ${}^{3}$	NS
Tear film break-up time (TBUT) (in seconds)	11.9 ± 0.96 s	12.6 s	11.8 ± 1.1 s	12.1 ± 0.7 s	NS
Axial length	24.13 ± 0.7 mm	24.01 ± 0.4 mm	23.47 ± 0.7 mm	23.13 ± 0.6 mm	NS
Contrast sensitivity (by FACT chart)	1.42 ± 0.12	1.423 ± 0.11	1.373 ± 0.09	1.37 ± 0.94	NS
D, diopter; NS, not significant					

Corneal flattening by 1.5 D in mean K (3 mm zone) and 1.2 D (5 mm zone) was observed in OK group patients by the end of the study period. The 5-mm zone keratometry documented the flattening earlier, beginning at 7 days compared to the 14 days for the 3-mm zone keratometry map.

During the entire follow-up period, perceived lens tolerance and comfort were high, with no dropouts seen. There were no statistically significant changes in TBUT between the baseline and four-month follow-up measurements in either group – 11.9 ± 0.96 s and 11.8 ± 1.1 s, respectively, in the OK group (*P* = 0.112) and 12.3 ± 0.6 s and 12.1 ± 0.7 s, respectively, in the control group (*P* = 0.132). Intergroup comparison revealed no statistically significant difference between the two groups in TBUT values measured before intervention (*P* = 0.90) or after four months (*P* = 0.123). This implied that no significant tear film disruptions were observed at any of the follow-ups in either the study group or the control group.

Contrast sensitivity (CS) was measured using FACT charts at 3 meters. There were no statistically significant differences in contrast sensitivity, as indicated by pre- to post lens prescription CS values, for either the OK (pre: 1.4 ± 0.1, post: 1.4 ± 0.1) (*P* = 0.189) or CL (pre: 1.37 ± 0.1, post: 1.37 ± 0.9) (p = 1.0) groups. The change in contrast sensitivity in the OK group (0.4 grid) was not significant as compared to no change in the CL group patients during the entire follow-up period of four months.

Subjective improvements in vision quality in the eyes treated with the OK lenses were adequate for the performance of daily activities with no additional vision aids. Subjective score assessments improved in all patients at all follow-ups, with only a handful of patients complaining of ghosting of images, glare, or difficulties in dim light on day 1. These complaints resolved by the end of four months. No significant lens decentration, corneal abrasion, dry eye, or sight-threatening complications such as microbial keratitis was noted in any of the subjects over the entire follow-up.

##  DISCUSSION

Orthokeratology utilizes contact lenses to “reduce, modify, or eliminate refractive errors.”^[14]^ A resurgence of interest in this technology occurred after the advent of reverse-geometry lens designs and use of an extremely high Dk material which makes these lenses easy to center and safer for the cornea. Reverse-geometry OK lenses incorporate a secondary curve, steeper than the lens base curve, to aid centration and are fitted with a base curve flatter than the central corneal curvature. This applies pressure to the central treatment zone, altering the corneal shape from prolate to oblate after overnight wear.^[15]^ OK is reportedly effective in slowing myopia progression over a 12-year follow-up period with a clinically acceptable safety profile.^[14]^

In our study, 60% of refractive correction was achieved after the first overnight OK wear, and almost 100% visual correction was achieved after one week of continuous overnight OK lens wear. This dramatic initial visual improvement is consistent with previous studies.^[15,16]^ The initial correction achieved in the morning after overnight OK-wear starts wearing off within hours due to the regression of the corneal shape, leading to visual blurring in the afternoon period.^[13]^ This fact was confirmed by our results, with patients complaining of afternoon blur with a drop of 1.2 Snellen lines in the evening during the first week. This effect is usually transient and resolves over a week.^[17]^ Stabilization of visual gain varies, with most studies reporting a gestation of four weeks and others—like ours—a period of 7–10 days.^[15,16][18][19][20]^

A concern with OK lenses is loss of contrast; however, in this series contrast sensitivity (and visual acuity) did not deteriorate either in the OK group or the control group after lens wear.^[22]^ A study by Tang et al measuring contrast sensitivity under both photopic and mesopic conditions corroborated this finding.^[23]^

Corneal flattening by a mean of 1.2 D was observed by four months, with maximum flattening after initial overnight use. This correlated more with the 5-mm zone topography value, which also picked up the peripheral changes earlier. These discrepancies in the 3- and 5-mm zones can be explained by the principle of OK where the lens hydraulically redistributes epithelial cells from the center toward the periphery and induces central corneal thinning and mid-peripheral thickening.^[13]^ Thus, 5-mm zone keratometry is a more sensitive indicator of OK-induced change and detects these changes earlier than the 3-mm zone map.

The most common problem associated with OK lens use is inadequate lens centration with superior displacement due to Bell's phenomenon during sleep, causing with-the-rule astigmatism.^[11]^ Sun et al have recently reported increased corneal irregularity and ocular HOA (spherical aberrations and comas) with OK lenses, despite improvements in refractive error.^[24]^ No significant lens decentration was seen in our patients.

In our series, no case of induced toxicity was noted, and no patient presented with any evidence of corneal damage or infiltration. This could be a result of the explicit lens care instructions that were given, and the vigilance of our motivated patients in carrying out lens hygiene measures. A review of literature on infectious keratitis associated with OK lens use reports that *Pseudomonas aeruginosa *and *Acanthamoeba *are the most commonly identified infectious agents with the majority of infections resulting in corneal scar formation, necessitating surgical intervention in 10% of eyes.^[25]^ Previous studies have emphasized the importance of proper lens care and maintenance of lens hygiene to ensure the safety of long-term OK lens wear.^[26,27]^

This study was designed as a pilot study evaluating efficacy and safety of OK, and longer follow-ups are necessary to establish the long-term effects of OK lenses. As the risk of keratitis is not high, substantial, long-term follow-up cohorts are necessary to evaluate the risk of keratitis associated with these contact lenses. No lens is considered completely safe for overnight wear. In addition, given that refractive surgeries such as LASIK/SMILE are very popular, some may question the relevance of OK lenses.^[28]^ However, OK lenses are non-invasive, reversible, and relatively safe to use (even in thin corneas) and hence stay relevant, especially in a developing country like ours.

In conclusion, OK is an effective and safe method for correcting moderate myopia in motivated young adults. Use of OK lenses was not associated with deleterious corneal complications, even in wearers residing in semi-tropical environments.

##  Acknowledgments

The authors would like to thank Dr. Mamtamayi Priyadarshani (Prkamya Visions) for her technical support and guidance.

##  Financial Support and Sponsorship

Nil.

##  Conflicts of Interest

There are no conflicts of interest.
